# Albumin-to-alkaline phosphatase ratio as a promising indicator of prognosis in human cancers: is it possible?

**DOI:** 10.1186/s12885-021-07921-6

**Published:** 2021-03-08

**Authors:** Lin An, Wei-tian Yin, Da-wei Sun

**Affiliations:** 1 Department of Hand Surgery, China-Japan Union Hospital of Jilin University, Changchun, 130033 Jilin China; 2 Department of Hepatobiliary and Pancreatic Surgery, The First Hospital of Jilin University, Changchun, 130021 Jilin China

**Keywords:** Albumin-to-alkaline phosphatase ratio (AAPR), Cancers, Prognosis, Meta-analysis

## Abstract

**Background:**

The impact of albumin-to-alkaline phosphatase ratio (AAPR) on prognosis in cancer patients remains uncertain, despite having multiple relevant studies in publication.

**Methods:**

We systemically compiled literatures from 3 databases (*Cochrane Library, PubMed,* and *Web of Science*) updated to May 24th, 2020. Hazard ratios (HRs) and 95% confidence intervals (CIs) were computed and synthesized using STATA 14, values were then pooled and utilized in order to assess the overall impact of AAPR on patient’s prognosis.

**Results:**

In total, 18 studies involving 25 cohorts with 7019 cases were incorporated. Pooled results originated from both univariate and multivariate analyses (*HR = 2.14, 95%CI:1.83–2.51, random-effects model; HR = 1.93, 95%CI:1.75–2.12*, *fixed-effects model*; respectively) suggested that decreased AAPR had adverse effect on overall survival (OS). Similarly, pooled results from both univariate and multivariate analysis of fixed-effects model, evinced that decreased AAPR also had adverse effect on disease-free survival (DFS) (*HR = 1.81, 95%CI:1.60–2.04, I*^*2*^ *= 29.5%, P = 0.174; HR = 1.69, 95%CI:1.45–1.97, I*^*2*^ *= 13.0%, P = 0.330*; respectively), progression-free survival (PFS) (*HR = 1.71, 95%CI:1.31–2.22, I*^*2*^ *= 0.0%, P = 0.754; HR = 1.90, 95%CI:1.16–3.12, I*^*2*^ *= 0.0%, P = 0.339*; respectively), and cancer-specific survival (CSS) (*HR = 2.22, 95%CI:1.67–2.95, I*^*2*^ *= 5.6%, P = 0.347; HR = 1.88, 95%CI:1.38–2.57, I*^*2*^ *= 26.4%, P = 0.244*; respectively). Admittedly, heterogeneity and publication bias existed, but stratification of univariate meta-analytic results, as well as adjusted meta-analytic results via trim and fill method, all showed that AAPR still significantly correlated with poor OS despite of confounding factors.

**Conclusions:**

In summary, decreased AAPR had adverse effect on prognosis in cancer patients. As an inexpensive and convenient ratio derived from liver function test, AAPR might become a promising indicator of prognosis in human cancers.

**Supplementary Information:**

The online version contains supplementary material available at 10.1186/s12885-021-07921-6.

## Background

Cancer is a major public health problem worldwide and is the second leading cause of death in the United States [1]. According to GLOBOCAN 2018, an estimated 18.1 million new cancer cases and 9.6 million cancer deaths happened worldwide [2]. Due to the growing and aging population as well as advances in diagnosis and therapy, cancer survivors number continues to increase [3]. So far, the prognostic markers for cancer survivors are diverse, but most of them except clinical-pathological factors, are not used in our clinical work due to high cost or inconvenience. Therefore, seeking practical markers to assess patients’ prognosis before the administration of treatment is urgently needed, so that therapeutic modality could be individually tailored, or augmented for an improved outcome [4].

Liver function test (LFT) is an universally used laboratory test to assess liver function in clinical work. Serving as an indicator of LFT, albumin is the most abundant protein in human plasma, and its concentration reflects the protein status of the blood and function of internal organs. Meanwhile, albumin is also a valuable biomarker of diverse diseases, including both malignant tumors and benign diseases (*liver diseases, inflammation, malnutrition, and diabetes mellitus,* etc.) [5, 6]. Alkaline phosphatase (ALP) is another important indicator of LFT, whose elevation is universally recognized as a marker of hepatobiliary or skeletal diseases [7]. Besides, the elevation of ALP was also reported to be found in diverse malignancies (*osteosarcoma, testicular neoplasm, prostate cancer, pancreatic cancer, breast cancer, and ovarian cancer,* etc.), and its elevation was usually correlated with poor outcomes [8]. Interestingly, as a combined ratio index derived from LFT, albumin to alkaline phosphatase ratio (AAPR) was firstly investigated to be a novel index of prognosis in hepatocellular carcinoma (HCC) patients in 2015 [9]. Henceforth, a series of studies have tried to explore the use of AAPR as a marker of prognosis in human cancers [10–26].

However, results from these emerging findings are inconsistent. For instance, some of them evinced that elevated AAPR was associated with poor survival outcomes [9, 10, 12–21, 23–26], but others evinced that elevated AAPR was not correlated with survival outcomes [11, 22]. Additionally, qualities of the above mentioned studies are variable, especially in terms of cancer types and methodology. Therefore, this meta-analysis was conducted to determine whether AAPR can serve as a novel indicator of prognosis in human cancers.

## Methods

This meta-analysis was conducted in accordance with *Preferred Reporting Items for Systemic Reviews and Meta-analysis (PRISMA)* issued in 2009 [27].

### Literatures research

We systemically sought relevant literatures in 3 databases (Cochrane Library, PubMed, and Web of Science). The search strategies were “*albumin-to-alkaline phosphatase ratio in All Text OR albumin to alkaline phosphatase ratio in All Text OR AAPR in All Text*” used in Cochrane Library, “*(((albumin-to-alkaline phosphatase ratio) OR AAPR) OR albumin to alkaline phosphatase ratio OR Albumin/alkaline Phosphatase Ratio)*” used in PubMed, *TS = (“albumin-to-alkaline phosphatase ratio” OR “AAPR” OR “albumin to alkaline phosphatase ratio” OR “Albumin/alkaline Phosphatase Ratio”)* used in Web of Science. The searching time without starting time updated to May 24th, 2020. During the process of full-text reading, the references of retrieved literatures were further manually browsed to find underlying literature.

### Criteria for literature selection

In this process, we selected the literatures based on three major criteria. First, the clinical study investigated the prognostic determinant role of AAPR regardless of human cancer type. Secondly, the survival endpoints of mentioned cancers are required to be well documented, including overall survival (OS), recurrence-free survival (RFS), disease-free survival (DFS), progression-free survival (PFS) and/or cancer-specific survival (CSS). Lastly, the hazard ratios (HRs) of the study endpoints should either be original or be calculated by utilizing the data, tables or graphs provided in the literature. We discarded case reports, review articles and comment letters. In situations where multiple literature used data from the same population sources, we preferred the literature with the maximum amount of cases.

### Data extraction and quality assessment

Two authors (AL and SDW) generated a compiled table, including the following contents: 1st author, year of publication, cancer site, sample size, study duration, design approach, cutoff value of AAPR, number of cases in each group, treatment strategy, survival outcome, HR data, analytic method (univariate/multivariate), HR source and follow-up interval. If the HR was not raw, the software Engauge Digitizer 4.1 was used to read the Kaplan-Meier curve to estimate the number of deaths/recurrences/survivors. Then, HR with its 95% CI was estimated by following practical methods (incorporating summary time-to event data into meta-analysis) created by Tierney et al. [28]. The authors extracted the contents of the table individually, and then exchanged their results. In this meta-analysis, *Newcastle-Ottawa Scale (NOS)* score was utilized to assess the included literature quality [29]. During this process, any discrepancies of opinion were resolved by reaching consensus through meetings held by participating authors.

### Statistic analysis

Software STATA 14 was utilized for data analysis in this research. HRs and 95% CIs were combined to evaluate the overall impact of AAPR on prognosis, including OS, DFS, PFS and CSS. If the lower limit of pooled 95% CIs was greater than 1, the decreased AAPR was considered to have an adverse effect on prognosis. Heterogeneity across included studies was examined by Chi-square test and *I*^*2*^, and the significance was set at either or both of *P < 0.1* and *I*^*2*^ *> 50%*. In case of both *I*^*2*^ *< 50%* and *P ≥ 0.1*, we used a fixed-effects model to compute the pooled HRs. Otherwise, the random-effects model was adopted to perform data analysis. In addition, we assessed publication bias by following *Begg’s* and *Egger’s* methods [30, 31]. We followed a *trim and fill* method to further testify the stability of pooled results, when cases of publication bias were found [32]. Across this research, a *P* value that is less than *0.05* was deemed as significant. In this research, we followed the methodology of statistic analysis, which was also used in our previously published research [33].

## Results

### Summary of systemic literature search

**Fig. 1:**
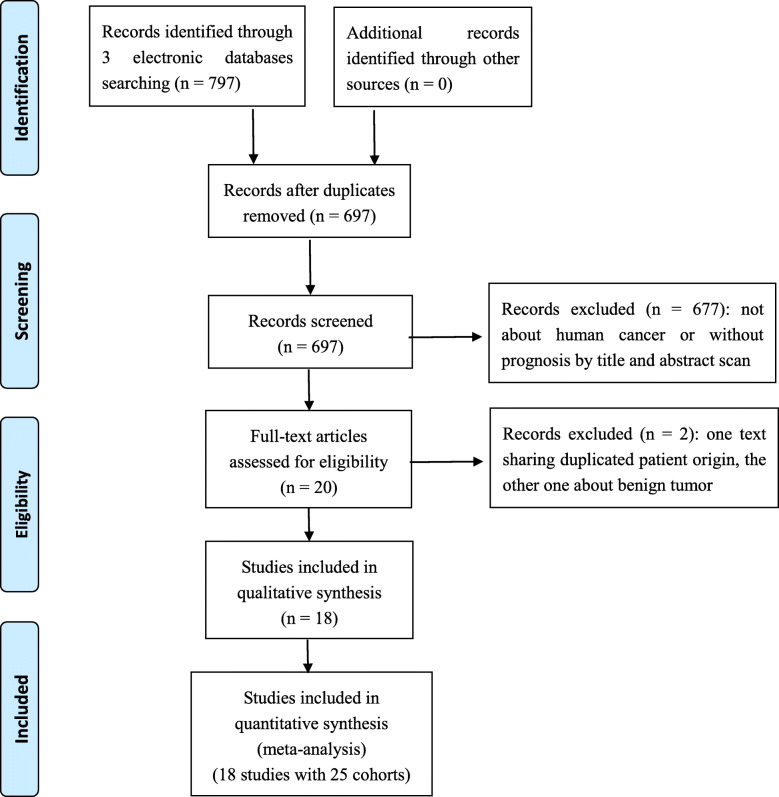
The literature search process used in this meta-analysis

As shown in Fig. 1 by following the PRISMA flow chart [27], 18 studies with 25 cohorts were incorporated in our meta-analysis finally [9–26]. The publishing year for these studies ranged from 2015 to 2020, with the total number of cancer cases of 7019, all of which were based in Asian countries (16 from China's mainland, the other two from Korea and Hong Kong). In terms of cancer sites, 5 studies reported lung cancer (LC), 4 studies reported HCC, 3 studies reported nasopharyngeal carcinoma, and the other 6 studies reported cervical cancer, cholangiocarcinoma, breast cancer, renal cell cancer, upper tract urothelial carcinoma, pancreatic duct adenocarcinoma, respectively. All other basic information relevant to these studies are displayed in Table 1. Following the NOS criteria, all of the included studies achieved the score ≥ 6, with the score ranging from 6 to 8 (Supplementary table).

**Table 1:** Summary items of included studies in this meta-analysis

1st author (Ref.)	Year	Country	Cancer site	Sample size	Study duration	Study design	AAPR cutoff value	High	Low	Treatment strategy	Survival outcome	HR (95%CI)	Source (Via analysis)	Follow-up (months)
Li H [10] *Training*	2020	China	HCC	149	2003–2014	R	0.38 by ROC	72	77	LT	OS	1.98 (1.28–3.08)	Crude (U)	More than 60
											OS	1.71 (1.06–2.71)	Crude (M)	
											DFS	1.11 (0.69–1.76)	Estimated by Curve (U)	
Li H [10] *Validation*	2020	China	HCC	61	2003–2014	R	0.38 by ROC	26	35	LT	OS	2.72 (1.29–5.75)	Estimated by Curve (U)	More than 60
											DFS	1.80 (0.88–3.70)	Estimated by Curve (U)	
Zeng X [11]	2020	China	NPC	255	2014–2018	R	0.63 by ROC	101	154	Multiple modalities	OS	1.57 (0.67–3.72)	Crude (U)	Median 33.5 (2.1–151.2)
											DFS	1.98 (0.94–4.14)	Crude (U)	
Zhou S [12]	2020	China	SCLC	224	2009–2018	R	0.35 (NR)	171	53	Chemotherapy	OS	1.55 (1.07–2.25)	Crude (U)	Up to 90
											OS	1.65 (1.11–2.46)	Crude (M)	
Li Q [13]	2020	China	HCC	188	2010–2015	R	0.40 by X-tile	94	94	Curative resection	OS	1.80 (1.30–2.52)	Estimated by Data (U)	Median 46.5
											DFS	1.57 (1.21–2.04)	Estimated by Data (U)	
Zhang C [14]	2019	China	CC	230	2008–2014	R	0.68 by ROC	89	141	Curative resection	OS	2.97 (1.23–7.19)	Crude (U)	Median 80 (12–137)
											OS	3.02 (1.24–7.41)	Crude (M)	
											DFS	2.49 (1.15–5.41)	Crude (U)	
											DFS	2.58 (1.17–5.68)	Crude (M)	
Xia A [15] *Training*	2019	China	RCC	419	2004–2014	R	0.39 by ROC	365	54	Curative resection	OS	3.00 (1.62–5.57)	Estimated by Data (U)	Median 50.0 (30.4–83)
											OS	2.75 (1.27–5.95)	Crude (M)	
											CSS	2.43 (1.20–4.93)	Estimated by Data (U)	
											CSS	3.04 (1.28–7.24)	Crude (M)	
Xia A [15] *Validation*	2019	China	RCC	204	2004–2014	R	0.39 by ROC	179	25	Curative resection	OS	4.77 (2.16–10.52)	Estimated by Data (U)	Median 50.2 (29.8–83.1)
											CSS	4.48 (1.59–12.61)	Estimated by Data (U)	
Li SJ [16]	2019	China	LC	390	2013–2015	P	0.57 by ROC	178	212	Curative resection	OS	4.76 (2.56–8.33)	Crude (U)	Median 50.0 (12–66)
											OS	3.23 (1.67–6.25)	Crude (M)	
											DFS	2.17 (1.43–3.33)	Crude (U)	
											DFS	1.67 (1.06–2.63)	Crude (M)	
Xiong JP [17]	2019	China	CCA	303	2002–2014	R	0.41 by ROC	253	50	Multiple modalities	OS	3.56 (1.28–9.92)	Crude (U)	Median 21
											OS	2.88 (1.19–5.78)	Crude (M)	
											DFS	2.52 (1.38–4.75)	Crude (U)	
											DFS	2.31 (1.40–3.29)	Crude (M)	
Zhang L [18]	2019	China	NSCLC	496	2006–2010	R	0.64 by ROC	199	297	Curative resection	OS	2.15 (1.64–2.82)	Crude (U)	Median 47 (2–96)
											OS	1.87 (1.22–2.74)	Crude (M)	
											DFS	2.18 (1.66–2.85)	Crude (U)	
											DFS	1.96 (1.30–2.96)	Crude (M)	
Li D [19]	2019	China	NSCLC	290	2007–2013	R	0.36 by ROC	201	89	Multiple modalities	OS	1.70 (1.32–2.20)	Crude (U)	Median 16 (1–84)
											OS	1.53 (1.17–1.98)	Crude (M)	
Li X [20]	2019	China	SCLC	122	2013–2015	R	0.61 by ROC	37	85	Chemoradiotherapy	OS	1.69 (1.02–2.78)	Crude (U)	Up to 70
											PFS	1.61 (1.01–2.50)	Crude (U)	
Long ZQ [21]	2019	China	BC	746	2011–2013	R	0.525 by ROC	621	125	Curative resection	OS	2.78 (1.45–5.23)	Crude (U)	More than 60
											OS	2.24 (1.02–4.88)	Crude (M)	
Kim JS [22]	2019	Korea	NPC	100	1998–2016	R	0.487 by ROC	80	20	Chemoradiotherapy	OS	1.77 (0.74–4.24)	Crude (M)	Median 50.6
											PFS	1.40 (0.63–3.10)	Crude (M)	
Tan P [23]	2018	China	UTUCs	692	2003–2016	R	0.58 by ROC	249	443	Curative resection	OS	1.82 (1.37–2.42)	Crude (U)	Median 42
											OS	1.59 (1.19–2.13)	Crude (M)	
											DFS	1.55 (1.20–2.00)	Crude (U)	
											DFS	1.34 (1.03–1.74)	Crude (M)	
											CSS	2.03 (1.46–2.80)	Crude (U)	
											CSS	1.75 (1.25–2.44)	Crude (M)	
Chen ZH [24] *Training*	2018	China	HCC	372	2009–2013	R	0.439 by ROC	117	255	TACE	OS	1.26 (1.04–1.55)	Estimated by Curve (U)	More than 60
											OS	1.57 (1.16–2.12)	Crude (M)	
Chen ZH [24] *Validation 1*	2018	China	HCC	202	2009–2013	R	0.439 by ROC	NR	NR	Supportive care	OS	2.14 (1.17–3.90)	Crude (M)	More than 60
Chen ZH [24] *Validation 2*	2018	China	HCC	82	2013–2014	R	0.439 by ROC	NR	NR	TACE	OS	2.87 (1.25–6.29)	Crude (M)	More than 60
Pu N [25] *Training*	2017	China	PDAC	220	2007–2016	R	0.46 by ROC	98	122	Curative resection	OS	1.82 (1.34–2.47)	Crude (U)	Median 15 (1–105)
											OS	2.09 (1.27–3.42)	Crude (M)	
Pu N [25] *Validation*	2017	China	PDAC	134	2007–2016	R	0.46 by ROC	35	99	Curative resection	OS	2.16 (1.35–3.43)	Crude (U)	Median 15 (1–105)
											OS	2.18 (1.04–4.53)	Crude (M)	
Nie M [26]	2017	China	NPC	209	2008–2011	R	0.447 by ROC	142	67	Chemotherapy	OS	2.87 (1.97–4.17)	Crude (U)	Median 16.6 (1–66.6)
											OS	3.27 (1.71–6.25)	Crude (M)	
											PFS	1.76 (1.27–2.42)	Crude (U)	
											PFS	2.30 (1.22–4.33)	Crude (M)	
Chan AW [9] *Training*	2015	HongKong	HCC	217	2001–2006	R	0.23 by ROC	199	18	Curative resection	OS	3.35 (1.97–5.70)	Crude (U)	Median 44.5 (0.1–160.7)
											OS	2.36 (1.35–4.10)	Crude (M)	
											DFS	2.46 (1.55–3.92)	Crude (U)	
											DFS	1.85 (1.16–2.96)	Crude (M)	
Chan AW [9] *Validation 2*	2015	HongKong	HCC	425	2007–2011	R	0.23 by ROC	200	225	Palliative treatment	OS	2.19 (1.78–2.68)	Crude (M)	Median 5.3 (0.1–62.6)
Chan AW [9] *Validation 1*	2015	HongKong	HCC	256	2006–2011	R	0.23 by ROC	241	14	Curative resection	OS	1.93 (1.06–3.50)	Crude (M)	Median 38.9 (0.1–95.4)
											DFS	1.58 (1.01–2.46)	Crude (M)	

**Abbreviations:** *CCA* cholangiocarcinoma, *NSCLC* non-small cell lung cancer, *LC* lung cancer, *SCLC* small-cell lung cancer, *NPC* nasopharyngeal carcinoma, *UTUCs* upper tract urothelial carcinomas, *PDAC* pancreatic ductal adenocarcinoma, *HCC* hepatocellular carcinoma, *RCC* renal cell cancer, *NPC* nasppharyngeal carcinoma, *CC* Cervical cancer, *BC* Breast cancer, *R* retrospective, *P* prospective, *NR* not reported, *ROC* receiver operating characteristic, *LT* Liver transplantation, *TACE* Transcatheter arterial chemoembolization, *OS* over-all survival, *DFS* disease-free survival, *PFS* progression-free survival, *CSS* cancer specific survival, *HR* hazard ratio, *CI* confidence interval, *U* univariate analysis, *M* multivariate analysis

### Meta-analysis with OS

**Fig. 2:**
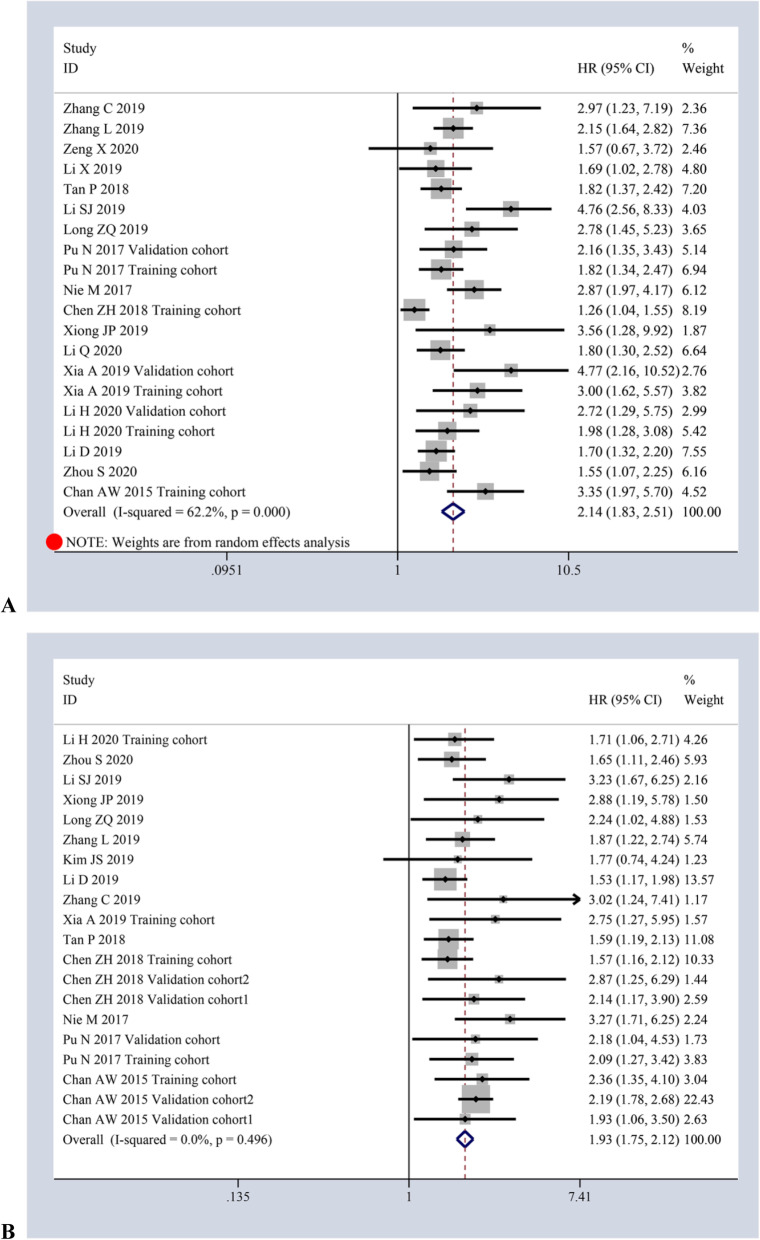
Forest plots of HR for AAPR and OS via univariate analysis (**a**) and multivariate analysis (**b**)

Regarding OS, 17 studies involving 20 cohorts with 5921 cases by univariate analytic results and 15 studies involving 20 cohorts with 6156 cases by multivariate analytic results were collected in total. It showed that the decreased AAPR had an adverse effect on OS in patients with cancers, which is not only taken from pooled univariate analytic results (*HR = 2.14, 95%CI:1.83–2.51, P ≤ 0.001*) of random-effects model (*I*^*2*^ *= 62.2%, p ≤ 0.001*) [Fig. 2a], but also from pooled multivariate analytic results (*HR = 1.93, 95%CI:1.75–2.12, P ≤ 0.001*) of the fixed-effects model (*I*^*2*^ *= 0.0%, p = 0.496*) [Fig. 2b].

### Meta-analysis with DFS

**Fig. 3:**
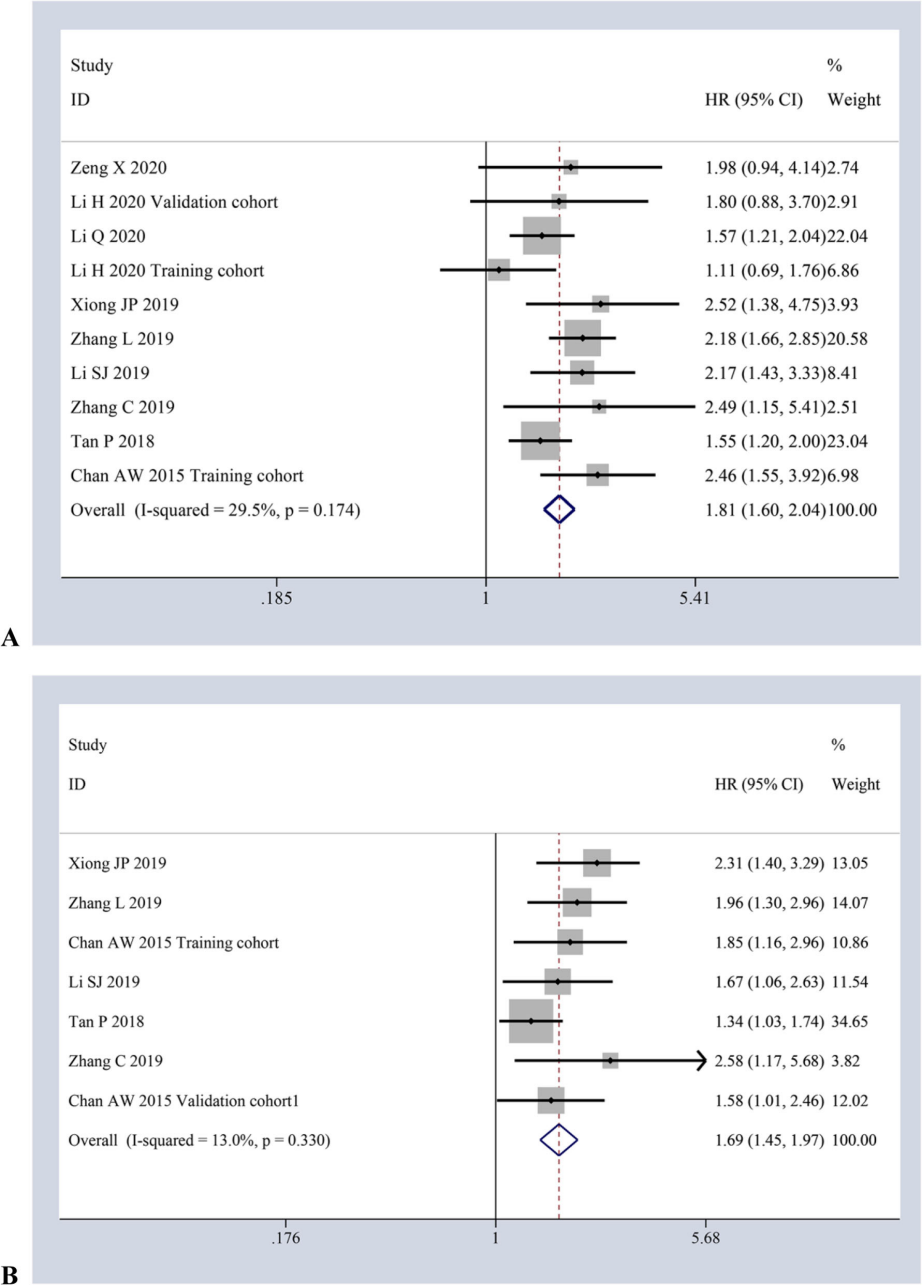
Forest plots of HR for AAPR and DFS via univariate analysis (**a**) and multivariate analysis (**b**)

There were a total of nine studies involving 10 cohorts with 3014 cases and six studies involving 7 cohorts with 2584 cases separately investigating the impact of AAPR on DFS via univariate and multivariate analysis. Heterogeneity existed in neither of these two pooled meta-analyses (*I*^*2*^ *= 29.5%, p = 0.174*; *I*^*2*^ *= 13.0%, p = 0.330;* respectively), revealing that decreased AAPR also had adverse effect on DFS (*HR = 1.81, 95%CI:1.60–2.04, P ≤ 0.001*; *HR = 1.69, 95%CI:1.45–1.97, P ≤ 0.001;* respectively) by the fixed-effects model [Fig. 3a-b].

### Meta-analysis with PFS and CSS

**Fig. 4:**
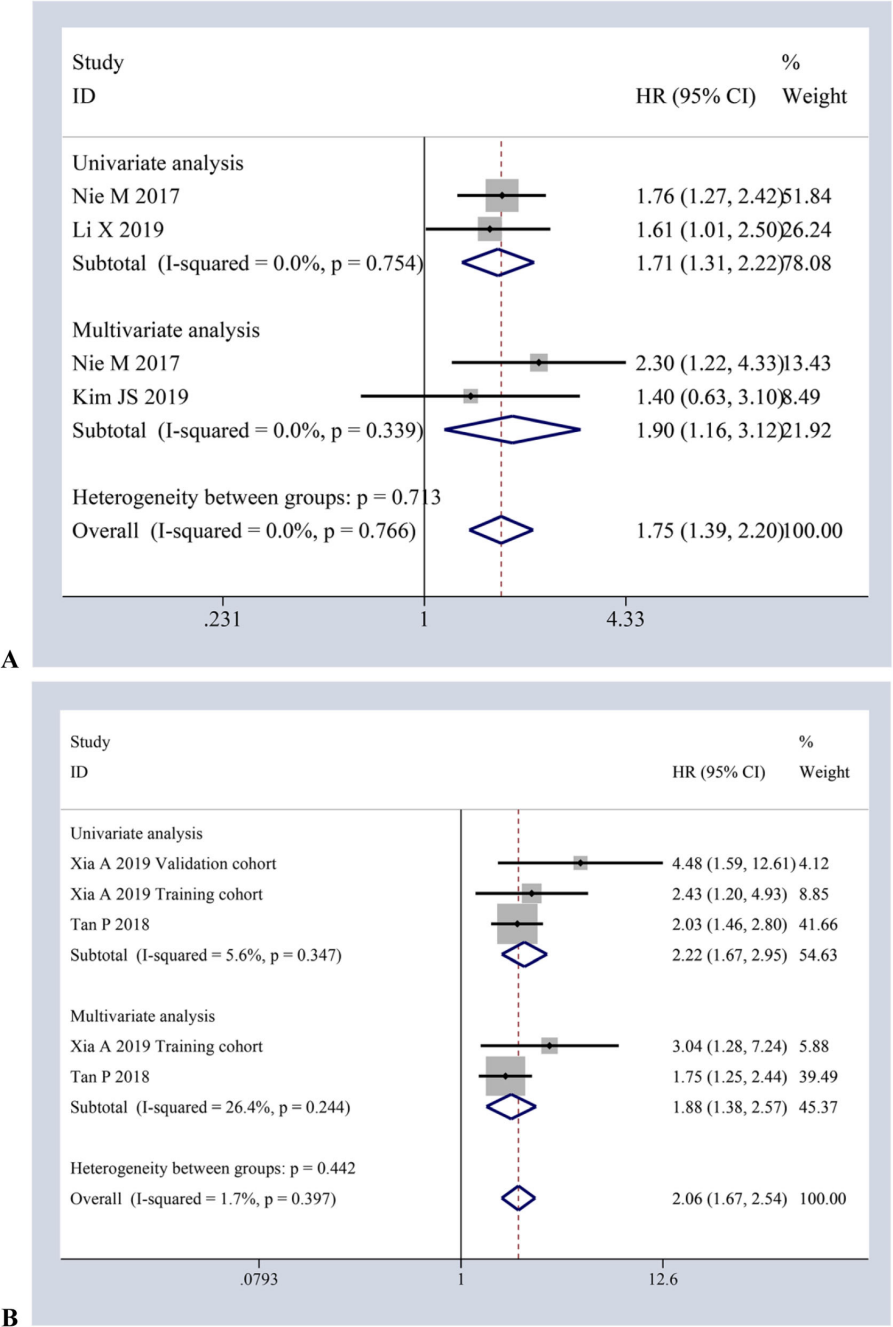
Forest plots of HR for the impact of AAPR on PFS (**a**) and CSS (**b**) via both univariate and multivariate analytic analysis

On the whole, two studies with 331 cases and two studies with 309 cases was separately used to evaluate the impact of AAPR on PFS by univariate and multivariate analysis, respectively. The pooled meta-analysis results of the fixed-effects model (*I*^*2*^ *= 0.0%, p = 0.754*; *I*^*2*^ *= 0.0%, p = 0.339;* respectively) supported that decreased AAPR also corelated with poor PFS (*HR = 1.71, 95%CI:1.31–2.22, P ≤ 0.001; HR = 1.90, 95%CI:1.16–3.12, P ≤ 0.001*) [Fig. 4a]. Similarly, two studies with 3 cohorts involving 1315 cases and two studies involving 1111 cases investigated the impact of AAPR on CSS via univariate and multivariate analysis, individually. According to the pooled results of the fixed-effects model (*I*^*2*^ *= 5.6%, p = 0.347*; *I*^*2*^ *= 26.4%, p = 0.244;* respectively), decreased AAPR also correlated with poor CSS (*HR = 2.22, 95%CI:1.67–2.95, P ≤ 0.001; HR = 1.88, 95%CI:1.38–2.57, P ≤ 0.001*) [Fig. 4b].

### Stratification for OS from univariate analysis

Heterogeneity existed across the included studies reporting OS via univariate analysis, thus, stratified meta-analysis was performed. These stratification were performed according to year of publication, cancer site, sample size, study design, treatment strategy, AAPR cut-off value, HR source and follow-up interval. Overall, it was found that the correlation between AAPR and OS remains stable despite the fluctuations of these variables, which was summarized in Table 2.

**Table 2:** Stratified analysis of OS meta-analysis results via univariate analysis

***Factor for stratification***	***No. of Cohorts***	***No. of Cases***	***No.of Low AAPR***	***N. of High AAPR***	***Pooled Data***			***Test for Heterogeneity***	
					***HR***	***95%CI***	***P value***	***I***^***2***^ ***(%)***	***P value***
***Overall calculation***	20	5921	2495	3426	2.14	1.83–2.51	*< 0.001*	62.2	0.000
***Year of publication***									
*After 2019*	14	4077	1491	2586	2.20	1.85–2.63	*< 0.001*	43.0	0.044
*Before 2019*	6	1844	1004	840	2.02	1.50–2.71	*< 0.001*	78.9	0.000
***Cancer site***									
*Liver cancer*	5	997	479	508	1.96	1.37–2.80	*< 0.001*	75.4	0.003
*Lung cancer*	5	1522	736	786	2.03	1.52–2.70	*< 0.001*	67.1	0.016
*Others*	10	3412	1280	2312	2.32	1.92–2.80	*< 0.001*	25.9	0.205
***Sample size***									
*≥ 224*	11	4417	1873	2544	2.06	1.64–2.58	*< 0.001*	69.2	0.000
*< 224*	9	1504	622	882	2.24	1.86–2.71	*< 0.001*	35.9	0.131
***Cut-off value for AAPR***									
*≥ 0.487*	7	2931	1457	1474	2.25	1.75–2.88	*< 0.001*	44.6	0.094
*< 0.487*	13	2990	1038	1952	2.10	1.72–2.56	*< 0.001*	67.0	0.000
***Study design type***									
*Prospective*	1	390	212	178	4.76	2.56–8.33	*< 0.001*	–	–
*Retrospective*	19	5531	2283	3248	2.05	1.77–2.37	*< 0.001*	55.8	0.002
***Treatment strategy***									
*Resection*	11	3936	1630	2306	2.20	1.95–2.50	*< 0.001*	47.9	0.038
*Others*	9	1985	865	1120	1.64	1.46–1.86	*< 0.001*	60.2	0.010
***HR source***									
*Crude*	16	5049	2287	2762	2.14	1.85–2.47	*< 0.001*	40.4	0.053
*Estimated*	4	872	208	664	2.21	1.42–3.44	*< 0.001*	79.2	0.001
***Follow-up interval***									
*≥ 5 years*	11	3385	1171	2214	2.36	1.81–3.06	*< 0.001*	70.1	0.000
*< 5 years*	9	2536	1324	1212	2.01	1.66–2.42	*< 0.001*	51.8	0.035

### Publication bias

**Fig. 5:**
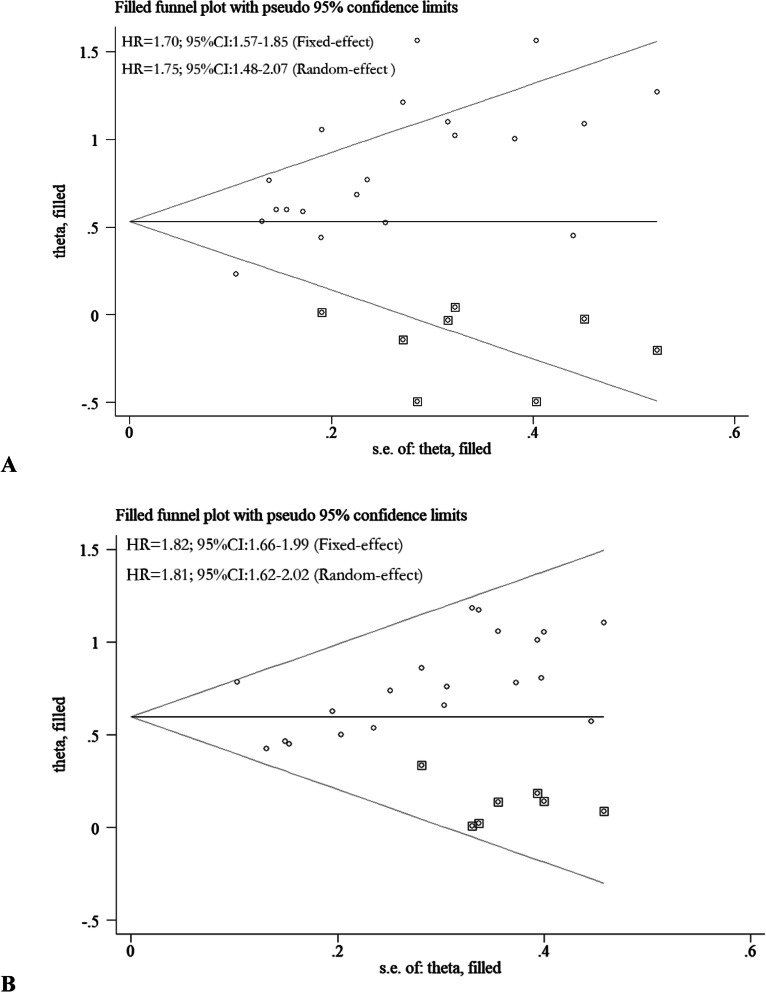
Adjusted meta-analysis results for OS via univariate analysis (**a**) and multivaritate analysis (**b**) by following the *trim* and *fill* method

Publication bias did not exist in meta-analysis with DFS via either univariate or multivariate analytic results (*P = 0.721, P = 0.382; P = 0.548, P = 0.148;* respectively), which was examined by following both *Begg’s* and *Egger’s* methods. But, publication bias existed in the meta-analysis with OS via both univariate and multivariate analytic results (*P = 0.021, P = 0.001; P = 0.018, P = 0.020*; respectively). Upon further investigation of the adjusted meta-analysis results by following the *trim* and *fill* method, AAPR is still significantly correlated with poor OS [Fig. 5a-b].

### Sensitivity analysis

**Fig. 6:**
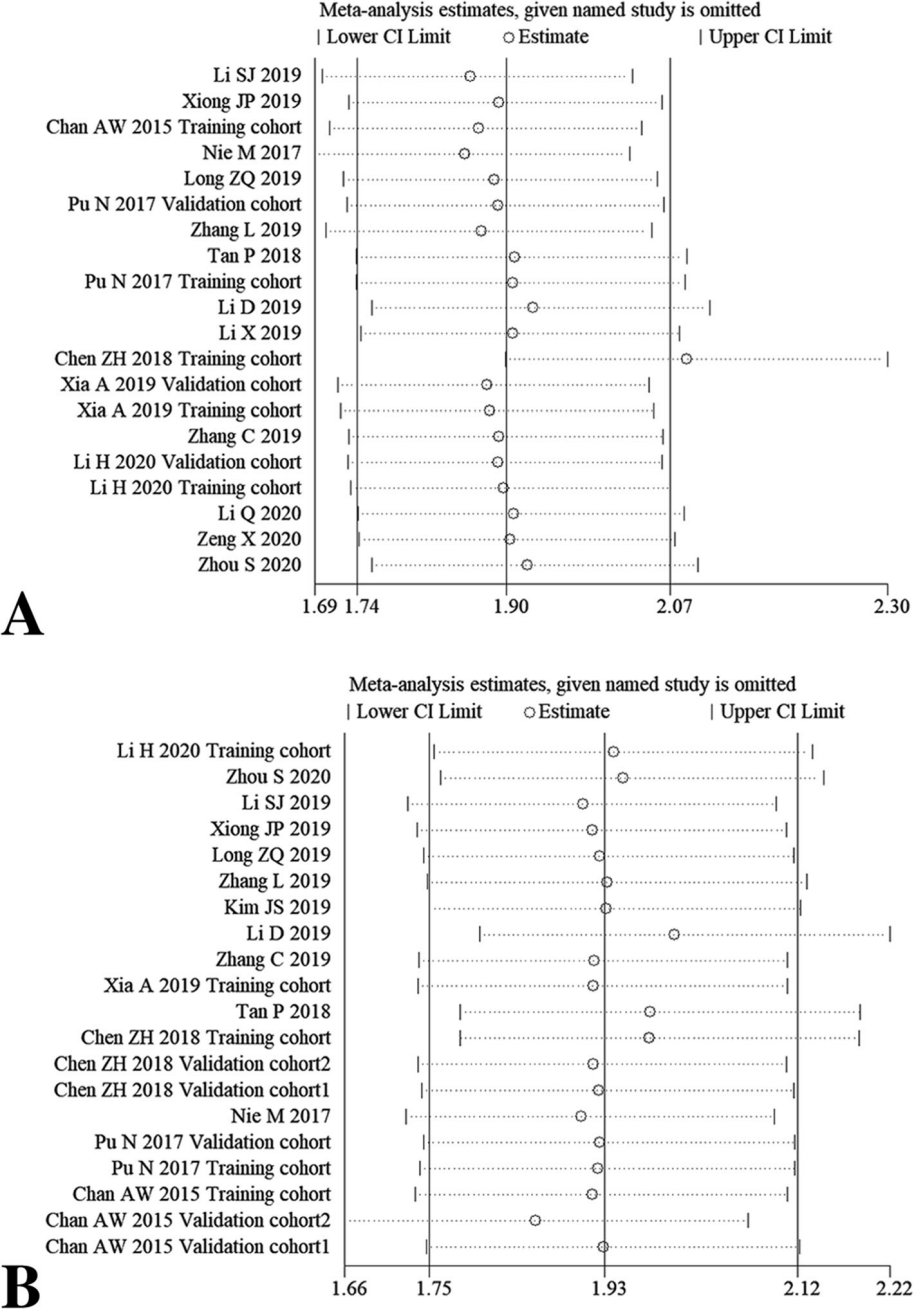

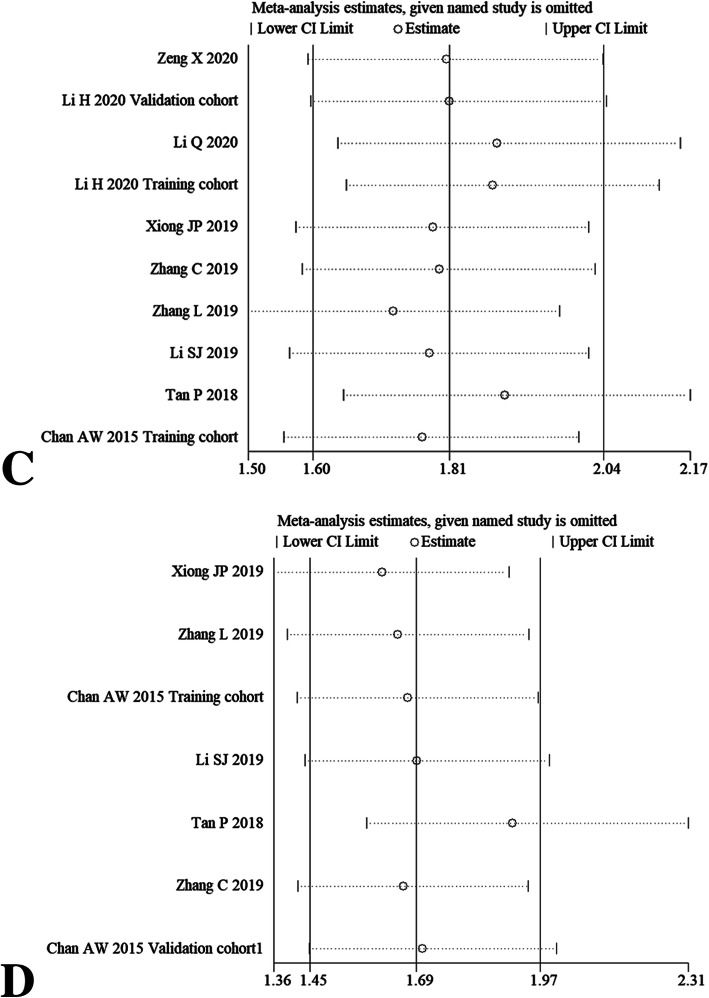

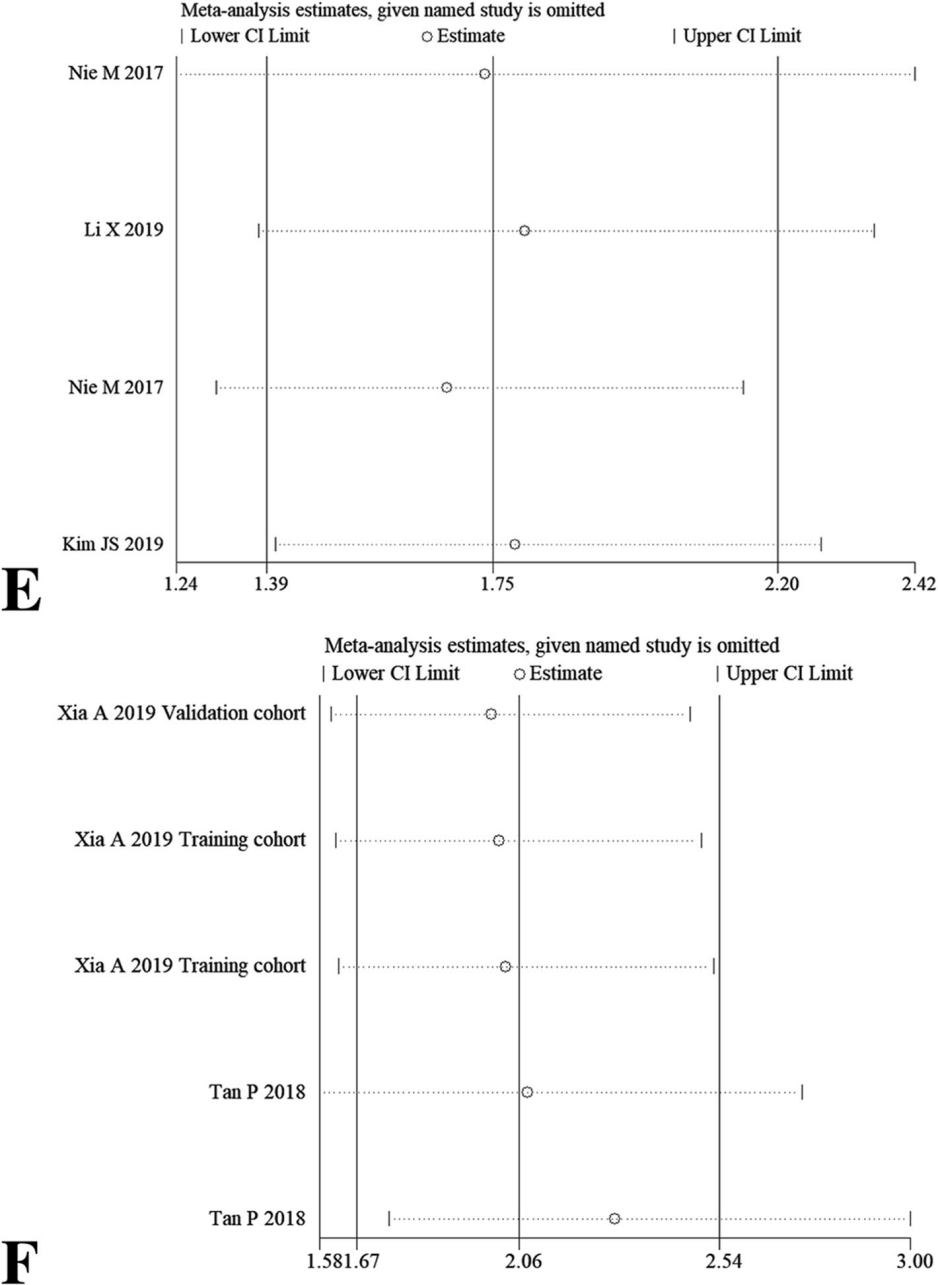
Sensitivity analysis for OS via univariate analytic results (**a**) and multivariate analytic results (**b**), DFS via univariate analytic results (**c**) and multivariate analytic results (**d**), PFS (**e**) and CSS (**f**) by omitting single included study

After omitting any individual study, we did not observe overall fluctuation of combined HRs for OS (Fig. 6a-b), DFS (Fig. 6c-d), PFS and CSS (Fig. 6e-f). Namely, the pooled HRs results from our meta-analysis were relatively robust.

## Discussion

It has been 5 years since the first reported study revealed that AAPR was a novel index of prognosis in patients with HCC [9]. In the years following, AAPR has been investigated to evaluate survival outcomes in diverse human cancers [10–26]. However, the association between AAPR and prognosis in patients with cancers has not been illustrated by evidence-based medicine yet. In this current research, we initially evaluated the prognostic role of AAPR in patients with cancers through meta-analysis. From the perspective of evidence-based medicine, our pooled meta-analysis results presented that decreased AAPR had adverse effects on OS, DFS, PFS and CSS in human cancers. In other words, decreased AAPR was associated with high mortality rate and high recurrence rate in cases with cancers.

In specific cases, both the univariate analytic results and the multivariate analytic results, were exploited and synthesized to evaluate the prognostic role of AAPR on OS/DFS/PFS/CSS. The combined meta-analysis results from these two analytic methods were unanimous. Heterogeneity is significant in OS via univariate analytic results, which might be due to the diverse clinicopathological factors, including patient’s characteristics, tumor classification, tumor stage, as well as follow-up interval. A stratified analysis was further conducted according to possible factors. Heterogeneity still existed in some of the subgroups, but as previously stated, the interaction between AAPR and OS remained stable. Publication bias was identified in the meta-analysis with OS via univariate and multivariate analysis, and therefore, trim and fill method was also further exploited to testify the association between AAPR and OS. Similarly, the adjusted meta-analysis results also demonstrated that AAPR significantly correlated with poor OS. In general, these results suggested that our meta-analysis results were comprehensive and convincing.

Albumin, the most abundant protein in plasma, is synthesized and secreted from the liver, and its concentration reflects the protein status of the blood and function of internal organs [6]. Hypoalbuminemia is frequently observed in human malignancies, which often serves as an indicator of poor nutritional status and also correlates with poor outcomes of cancer patients [6, 34]. Additionally, albumin has the physiological properties as an anti-oxidant and drug transporter, and therefore, hypoalbuminemia could cause the insufficiency of these functions, leading to poor postoperative outcomes [35]. Moreover, as a negative acute phase protein, albumin is associated with increased inflammatory status, whereupon increased inflammation usually leads to poor outcomes [35].

ALP comprising a heterogeneous group of enzymes, which are expressed and distributed in different human body tissues [36]. Accordingly, ALP can be categorized into tissue-specific and tissue nonspecific types. The tissue-specific type of ALP is only found in the intestine, placenta, and germinal tissue, whereas it can also secrete into circulation under specific stimulation. In contrast, the tissue-nonspecific ALP in the circulation (secreted by liver, bone, and kidneys) is gaining the interest of clinicians [36]. Use of ALP as a tumor marker can be dated back to the *1980*s [37]. From then on, hyperphosphatasia (Namely, elevated ALP level) has been proposed as prognostic indicator in various cancers, including prostate cancer [38], renal cell carcinoma [39], HCC [40], gastric cancer [41], pancreatic cancer [42], and osteosarcoma [43]. It has been previously proven that hyperphosphatasia is present in primary or metastatic cancer via increasing liver isoenzyme leakage, as well as causing local biliary obstruction [44]. Nevertheless, primary extrahepatic cancer does not necessarily have to involve the liver or bone, because some cancers present with paraneoplastic effect, resulting in liver isoenzyme leakage into serum (eg. renal cell carcinoma), and some rare cancers can also produce ALP (eg. Hodgkin lymphoma) [44].

The underlining mechanism behind AAPR becoming a prognostic indicator of human cancers should be due to the pathological properties of hypoalbuminemia and hyperphosphatasia. Indeed, the decrease of AAPR could be caused by either one or both of the two abnormalities, both of which significantly correlate with poor outcomes in human cancers. When compared with single indicator—hypoalbuminemia or hyperphosphatasia, AAPR might contribute to identifying more patients with poor prognosis, because some cases might present with normal serum albumin levels but hyperphosphatasia, or normal serum ALP levels but hypoalbuminemia. It’s well-known that both albumin and ALP are common serum biochemical indicators used during clinical work. Therefore, we inferred that AAPR can serve as a more practical and more comprehensive indicator of prognosis in human cancers. However, our meta-analysis results were based on available researches from Asian countries, the prognostic role of AAPR in cancers also needs to be assessed by further research in western countries, especially research conducted in the greater cancer community. Additionally, well-designed clinical diagnostic research based on large scale (*comparing the accuracy of AAPR, ALB, and ALP*), or meta-analysis based on diagnostic research is still warranted to clarify this issue.

Importantly, it should be noted that neither hypoalbuminemia nor hyperphosphatasia is cancer-specific. For instance, hypoalbuminemia could also be caused by malnutrition, as well as diverse benign diseases, such as liver disease, infectious disease, and nephrotic syndrome [45, 46]. Meanwhile, hyperphosphatasia is also involved in a variety of pathological processes not exclusive to cancer. This includes liver dysfunction (eg. bile duct obstruction), bone diseases (eg. bone formation after fracture) and endocrine diseases (eg. hyperparathyroid) [36]. Therefore, attention should be paid to these potential confounding factors when exploiting AAPR as potential prognostic marker in patients with cancers.

## Conclusions

In summary, decreased AAPR had adverse effects on prognosis in patients with cancers. As an inexpensive and convenient ratio derived from LFT, AAPR might become a promising indicator of prognosis in human cancers.

## Supplementary Information

**Additional file 1: Supplementary table.** The *Newcastle-Ottawa Scale (NOS)* scores for incorporated studies of this meta-analysis.

## Data Availability

Nearly all the data (HR with 95%CI) used in this meta-analysis were directly extracted from the 18 included studies originally, except 4 studies in which HR associated results were estimated according to Kaplan-Meier Curve or survival data.

## Abbreviations

AAPR: Albumin-to-alkaline phosphatase ratio
HR: Hazard ratio
CI: Confidence interval
OS: Overall survival
DFS: Disease-free survival
PFS: Progression-free survival
CSS: Cancer-specific survival
