# Supplementary material for: Albumin-to-alkaline phosphatase ratio as a promising indicator of prognosis in human cancers: is it possible?
Source: BMC Cancer. 2021 Mar 8;21:247. doi: 10.1186/s12885-021-07921-6 (PMC7938577; doi:10.1186/s12885-021-07921-6)
Supplement: Supplementary file 1 — Additional file 1: Supplementary table. The Newcastle-Ottawa Scale (NOS) scores for incorporated studies of this meta-analysis. [file 12885_2021_7921_MOESM1_ESM.docx]

**Supplementary table**. The Newcastle-Ottawa Scale (NOS) scores for incorporated studies of this meta-analysis.

| ***1^st^ author (Ref.)*** | ***Selection (score)*** | | | | ***Comparability (score)*** | ***Outcome (score)*** | | | ***Accumulated score*** |
| --- | --- | --- | --- | --- | --- | --- | --- | --- | --- |
|  | ***Exposed group*** | ***Nonexposed group*** | ***Exposure assessment*** | ***Interest before research*** |  | ***Outcome assessment*** | ***Follow-up interval (≥ 5 years)*** | ***Follow-up response*** |  |
| *Li H [10]* | 1 | 1 | 1 | 0 | 2 | 1 | 1 | 1 | 8 |
| *Zeng X [11]* | 1 | 1 | 1 | 0 | 1 | 1 | 0 | 1 | 6 |
| *Zhou S [12]* | 1 | 1 | 1 | 0 | 1 | 1 | 0 | 1 | 6 |
| *Li Q [13]* | 1 | 1 | 1 | 0 | 1 | 1 | 1 | 1 | 7 |
| *Zhang C [14]* | 1 | 1 | 1 | 0 | 2 | 1 | 1 | 1 | 8 |
| *Xia A [15]* | 1 | 1 | 1 | 0 | 2 | 1 | 1 | 1 | 8 |
| *Li SJ [16]* | 1 | 1 | 1 | 0 | 2 | 1 | 0 | 1 | 7 |
| *Xiong JP [17]* | 1 | 1 | 1 | 0 | 2 | 1 | 1 | 1 | 8 |
| *Zhang L [18]* | 1 | 1 | 1 | 0 | 2 | 1 | 1 | 1 | 8 |
| *Li D [19]* | 1 | 1 | 1 | 0 | 1 | 1 | 0 | 1 | 6 |
| *Li X [20]* | 1 | 1 | 1 | 0 | 1 | 1 | 0 | 1 | 6 |
| *Long ZQ [21]* | 1 | 1 | 1 | 0 | 1 | 1 | 1 | 1 | 7 |
| *Kim JS [22]* | 1 | 1 | 1 | 0 | 1 | 1 | 0 | 1 | 6 |
| *Tan P [23]* | 1 | 1 | 1 | 0 | 2 | 1 | 0 | 1 | 7 |
| *Chen ZH [24]* | 1 | 1 | 1 | 0 | 1 | 1 | 1 | 1 | 7 |
| *Pu N [25]* | 1 | 1 | 1 | 0 | 1 | 1 | 0 | 1 | 6 |
| *Nie M [26]* | 1 | 1 | 1 | 0 | 2 | 1 | 0 | 1 | 7 |
| *Chan AW [9]* | 1 | 1 | 1 | 0 | 2 | 1 | 1 | 1 | 8 |
